# Mitochondrial Functional Capacity Is Impaired in Angiotensin II-Infused Mice and Not Recovered by Metformin

**DOI:** 10.3390/biomedicines14040759

**Published:** 2026-03-26

**Authors:** Amanda Balboa Ramilo, Kevin Mani, Anders Wanhainen, Malou Friederich-Persson, Dick Wågsäter

**Affiliations:** 1 Department of Medical Cell Biology, Uppsala University, 751 23 Uppsala, Sweden; 2 Department of Surgical Sciences, Section of Vascular Surgery, Uppsala University, 751 85 Uppsala, Sweden; kevin.mani@uu.se (K.M.); anders.wanhainen@uu.se (A.W.); 3 Department of Diagnostics and Intervention, Surgery, Umeå University, 901 85 Umeå, Sweden

**Keywords:** abdominal aortic aneurysm, mitochondria, high-resolution respirometry

## Abstract

**Background:** The pathophysiological mechanisms of Abdominal Aortic Aneurysm (AAA) are not elucidated. Alterations in mitochondrial function, such as a reduction in oxidative phosphorylation (OXPHOS), have been observed at genome level and functionally in vascular smooth muscle cells. Metformin reduces AAA development and growth in diabetic patients, but the precise mechanisms are not known. In this paper we aim to demonstrate the feasibility of measuring mitochondrial functional capacity ex vivo in intact murine aneurysmal tissue and confirm a decrease in OXPHOS, and to determine if the protective effect of metformin on AAA is mediated by mitochondrial function. **Methods:** AAA was induced in ApoE KO mice by administration of angII (1000 ng/kg/min) through osmotic minipumps. Metformin was administered in drinking water at a dose of 100 mg/kg/day. The abdominal aorta was isolated in situ and mitochondrial functional capacity was analyzed ex vivo in whole permeabilized tissue by high-resolution respirometry. **Results:** Mitochondrial respiration was successfully measured ex vivo in whole aneurysmal tissue. Mitochondrial function was impaired in angII-treated mice, with decreased fold change in Complex I and Complex I+II oxygen consumption, relative to basal levels. Complex II oxygen consumption was also decreased in angII-treated mice. Rescue treatment of mice with metformin did not affect or restore mitochondrial function. **Conclusions:** Mitochondrial function can be evaluated in murine whole aneurysmal tissue, providing a method for a physiological approach to the study of mitochondrial function in AAA. Mitochondrial function is impaired in AAA. However, rescue treatment with metformin is not sufficient to recover mitochondrial function and seems not to be the mechanism behind prevention of aneurysm.

## 1. Introduction

Abdominal Aortic Aneurysm (AAA) is a vascular disease characterized by degradation of the aortic wall, leading to enlargement and subsequent risk for rupture of the blood vessel. It affects <1% of non-smoking men over the age of 65 years, but up to 5% of smokers [1]. Although the exact pathophysiological mechanisms remain elusive, the process of aortic wall degradation in AAA formation involves inflammation and elastin degradation. Alterations in mitochondrial function have also been observed. In human and mouse vascular smooth muscle cells (VSMCs) there is a reduced expression of peroxisome proliferator-activated receptor gamma coactivator 1 alpha (*PGC1α*), which indicates an impairment of mitochondrial biogenesis [2]. On a functional level, reduction in mitochondrial respiration through oxidative phosphorylation (OXPHOS) is also observed. Genome analysis of human and mouse samples shows differential expression of OXPHOS-associated genes [3,4] and functional measurements of mitochondrial respiration in VSMC isolated from Fibulin-4 knockout (KO) mice [5], angiotensin II (angII)-induced AAA in apolipoprotein E (ApoE) KO mice and from human AAA [6] all demonstrate a lower oxygen consumption rate in AAA compared to healthy controls.

Most studies of mitochondrial function in AAA pathophysiology have been focused on VSMC. However, AAA is a complex disease, involving several cell types, including immune and endothelial cells. A more physiological approach, where mitochondrial function is assessed in the whole tissue, is necessary to understand its role in AAA pathogenesis. Mitochondrial functional capacity can be evaluated by high-resolution respirometry (HRR), a method that allows dynamic and integrative measurement of oxygen consumption by structurally intact mitochondria [7]. Evaluation of mitochondrial function in situ can be achieved by HRR, using permeabilized cells or tissues [8,9]. While mitochondrial function has been measured in murine aortic tissue [10,11,12,13], measurement in aneurysmal tissue has not been performed to date.

No pharmacological treatments are available for AAA. However, several epidemiological studies and meta-analyses demonstrate that treatment with metformin, a common treatment for type 2 diabetes mellitus (T2DM), reduces AAA development and growth in diabetic patients [14,15,16,17,18], which is confirmed in experimental AAA. In the elastase model, administration of metformin throughout the period of aneurysm induction and enlargement limits both AAA initiation and progression [19]. Administration of metformin four days after disease induction in this model also reduces AAA development [20]. In the angII model, treatment with metformin also reduced AAA initiation and progression [21,22,23]. Administration of metformin after angII induction of AAA has not been studied. The exact mechanism behind the inhibiting effect of metformin on AAA is not known but is partly explained by effects via AMPK signaling and PI3K/AKT/mToR pathway [22,23]. In pharmacological concentrations, metformin has shown to increase mitochondrial respiration [24]. Therefore, we hypothesize that metformin inhibits AAA development and progression by restoring the mitochondrial function of the aorta.

In this paper, we aim to demonstrate that it is possible to measure mitochondrial functional capacity ex vivo in intact aneurysmal tissue. Furthermore, we aim to confirm the predicted reduction in OXPHOS and determine if the protective effect of metformin on AAA development is mediated by restoration of mitochondrial function. Last, we aim to assess if treatment with metformin starting one week after aneurysm induction inhibits aneurysm development in the angII disease model.

## 2. Materials and Methods

### 2.1. AAA Development

Male, 8–10 weeks old, ApoE KO mice (*n* = 38) (Charles River, Sulzfeld, Germany) were used in the experiments and randomly divided into cages and treatments. Mice were kept in a 12:12 h light cycle, humidity- and temperature-controlled environment, with water and normal chow ad libitum. AAA was induced by subcutaneous implant of a model 1004 mini-osmotic pump (Alzet, Cupertino, CA, USA), releasing 1000 ng/kg/min of angII for 28 days. Control mice received a saline solution. Mice were treated with buprenorphine (0.1 mg/kg) twice daily for 48 hours postoperatively and mice were observed daily for any signs of pain or disability. If mice showed discomfort, pain or weight loss according to the local assessment for humane endpoint, the mice were sacrificed. In this study, no animals needed to be sacrificed due to reaching a humane endpoint.

**Figure 1:**
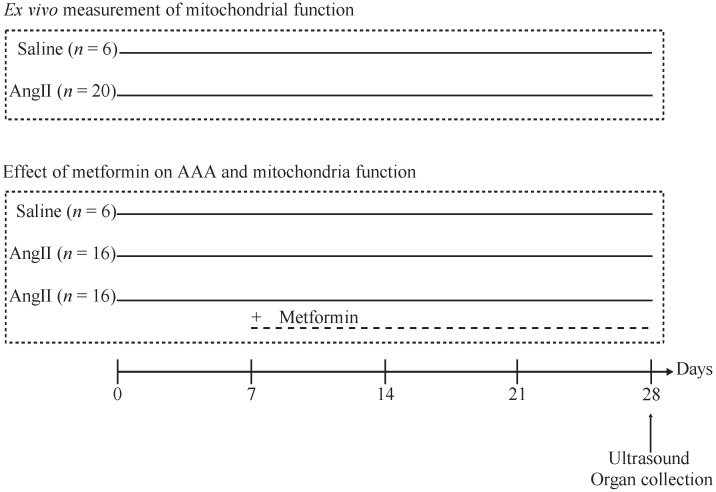
Graphical representation of Abdominal Aortic Aneurysm (AAA) induction and treatment scheme.

To determine if mitochondria function can be measured ex vivo in permeabilized mouse aortic tissue, mice were allocated to a control group (*n* = 6) and an angII-infused group (*n* = 20). To determine the effect of metformin on mitochondrial function, mice were allocated to a control group (*n* = 6), angII-infused group (*n* = 16) and an angII+metformin group (*n* = 16) (Figure 1). Metformin was administered in drinking water, at a dose of 100 mg/kg/day [21] starting seven days after disease induction (rescue treatment) (Figure 1).

Ex vivo measurement of mitochondrial functional capacity: to demonstrate the feasibility of measuring mitochondrial function in whole AAA tissue, AAA was induced in male ApoE KO mice (AngII, *n* = 20) by continuous infusion of angII 1000 ng/kg/min for 28 days, through a mini-osmotic pump. The control group (Saline, *n* = 6) received an infusion of saline solution for the same amount of time. On day 28 mice, aortic diameter was measured by high-frequency ultrasound. Mice were sacrificed by cervical dislocation and whole aortic tissue (supra and infrarenal) was collected for high-resolution respirometry analysis.

Effect of metformin on AAA and mitochondria functional capacity: to study the effect of metformin on AAA development, mice were allocated to one of the following three groups: saline (*n* = 6), angII (*n* = 16) or angII + met (*n* = 16). In this last group, treatment with metformin started seven days after disease induction with angII. Metformin was administered in drinking water, at a dose of 100 mg/kg/day. On day 28 mice, aortic diameter was measured by high-frequency ultrasound. Mice were sacrificed by cervical dislocation and whole suprarenal aortic tissue was collected for high-resolution respirometry analysis.

Aortic diameter at experimental endpoint was evaluated, under anesthesia (1.8% isoflurane, 100 mL/min), by high-frequency ultrasound (Vevo 1100, VisualSonics Inc., Toronto, ON, Canada). Measurements were performed at the point of largest diameter. AAA was defined as a diameter superior to 1.5 mm.

At termination, mice were euthanized by cervical dislocation and aortic tissue was collected. Suprarenal and infrarenal tissue were collected to assess the feasibility of the method. For evaluation of the effect of metformin, only suprarenal tissue was collected and evaluated.

The Uppsala Region animal ethics committee board, Sweden, approved all animal experiments (ethical approval number 5.8.18-15489/2022, date of approval 30 September 2022).

### 2.2. Analysis of Mitochondrial Respiration 

The abdominal aorta was isolated in situ, with the removal of surrounding connective and adipose tissue. Suprarenal aorta, spanning from the diaphragm to the renal arteries and infrarenal aorta, from the renal arteries to the iliac bifurcation, was collected separately. Aortic tissues were cut longitudinally, exposing the lumen of the vessel, blotted on a piece of paper and weighed. After weighing, samples were immediately placed in 1 mL of a 450 µg/mL digitonin solution, diluted in ice-cold isolation media A1, for 30 min, with shaking (50 rpm) at room temperature. Isolation media A1, prepared according to manual [25], contained 250 mM sucrose, 0.5 mM ethylenediaminetetraacetic acid disodium salt solution (Na_2_EDTA) and 10 mM Tris base, pH adjusted to 7.4. After permeabilization, aortic samples were washed in 1 mL ice-cold respiration media (Mir05) for 10 min. Mir05 was prepared according to manual [26] containing 0.5 mM ethylene glycol-bis(β-aminoethyl ether)-N,N,N′,N′-tetraacetic acid (EGTA), 3 mM MgCl_2_, 60 mM lactobionic acid, 20 mM taurine, 10 mM KH_2_PO_4_, 20 mM (4-2(hydroxyethyl)-1-piperazineethanesulfonic acid (HEPES), 110 mM sucrose and 1 g/L bovine serum albumin (BSA), pH 7.1. Samples were not allowed to dry between collection and analysis.

Immediately after washing, aortic tissues were transferred to the chamber of an oxygraph-2k (Oroboros Instruments, Innsbruck, Austria), where Mir05 was pre-warmed to 37 °C. To transfer the tissue sections into the oxygraph chamber, the stoppers were removed, the magnetic stirrer was paused, and the tissue was introduced with the help of a pipette tip. The stoppers were put back, and the magnetic stirrer was turned on for the system to re-equilibrate.

Previous to the placement of the aortic tissue into the oxygraph chamber, 5 µL pyruvate (2 M) and 10 µL malate (400 mM) were added to the chamber. Once the tissue was added and oxygen consumption stabilized, Complex I basal respiration (in the absence of ADP) was measured. The addition of 15 µL ADP (500 mM) followed, allowing measurement of Complex I-supported full respiration. Complex I+II-supported full respiration was evaluated after addition of 20 µL succinate (1 M). Addition of 1 µL rotenone (1 mM) allowed measurement of Complex II-supported full respiration. At the end of the analysis, stoppers were removed from the chamber and the magnetic stirrer turned off. Aortic tissue was removed from the chamber with the help of a pipette tip and immediately frozen in dry ice for further analysis.

All respiration measurements were normalized to the wet weight of the tissue sections. All chemicals were obtained from Sigma-Aldrich (St. Louis, MO, USA) and Scharlau Chemicals (Barcelona, Spain). Mitochondrial substrates and inhibitors were prepared according to the manual [27].

### 2.3. Statistical Analysis

Statistical analysis was performed with GraphPad Prism 10.1.2 software and R (version 4.1.1). Results are presented as mean ± standard deviation (SD). Sample size was calculated to identify changes of at least 50% between groups with a power of 80 and an α of 0.05. Shapiro–Wilk test was used to determine normal distribution.

Log-rank analysis was used for analysis of survival. Aortic diameter, suprarenal and infrarenal weight were compared by Mann–Whitney tests, when comparing two groups, and Kruskal–Wallis followed by Dunn’s test, when comparing three groups. Blood glucose and body weight were compared by ANOVA. Mitochondrial function parameters were analyzed by unpaired T-tests, when comparing two groups, or ANOVA followed by Tukey’s test when comparing three groups. Correlation between aortic diameter and oxygen consumption was performed by Pearson correlation analysis. *p* < 0.05 was considered statistically significant.

## 3. Results

### 3.1. AAA Development for Ex Vivo Measurement of Mitochondrial Functional Capacity

All control mice survived until the experimental endpoint. In the angII-treated animals, seven out of 20 mice died from dissection or rupture, in the first 6 days from disease induction. For the surviving mice, average aortic diameter was significantly higher in the angII-treated group (1.6 ± 0.44 mm), compared to control mice (1.1 ± 0.09 mm). The average wet weight of suprarenal and infrarenal aortas collected was significantly higher in the angII-treated group (11.1 ± 7.62 and 6.1 ± 1.14 mg) than in control mice (1.7 ± 0.80 and 3.0 ± 1.36 mg, Table 1). Wet weight of the suprarenal aorta correlated with the aortic diameter determined by ultrasound (r = 0.82, *p* < 0.05).

### 3.2. Mitochondrial Functional Capacity Is Impaired in Aortas of AngII-Treated Mice and Related to Aneurysm Size

**Figure 2:**
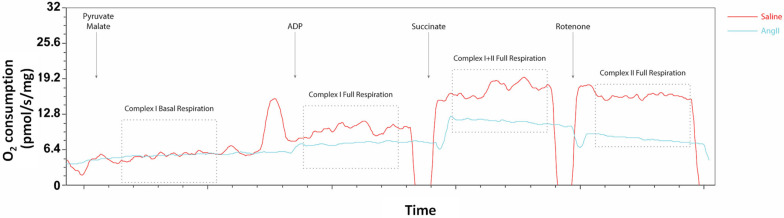
Representative curves for mitochondrial oxygen consumption in pmol/s/mg (normalized to tissue wet weight) vs. time in the saline group (red) and angII-infused group (light blue). Arrows represent the addition of substrate or inhibitor, and dotted boxes represent the parameters measured for evaluation of mitochondrial function. Complex I basal respiration measured after addition of pyruvate and malate; Complex I full respiration measured after the addition of ADP; Complex I+II full respiration measured after addition of succinate; and Complex II full respiration measured after addition of rotenone.

Mitochondrial respiration was successfully measured ex vivo in intact aneurysmal tissue, as observed by the representative curves of mitochondrial respiration in the suprarenal aorta of saline and angII-treated mice presented in Figure 2.

**Figure 3:**
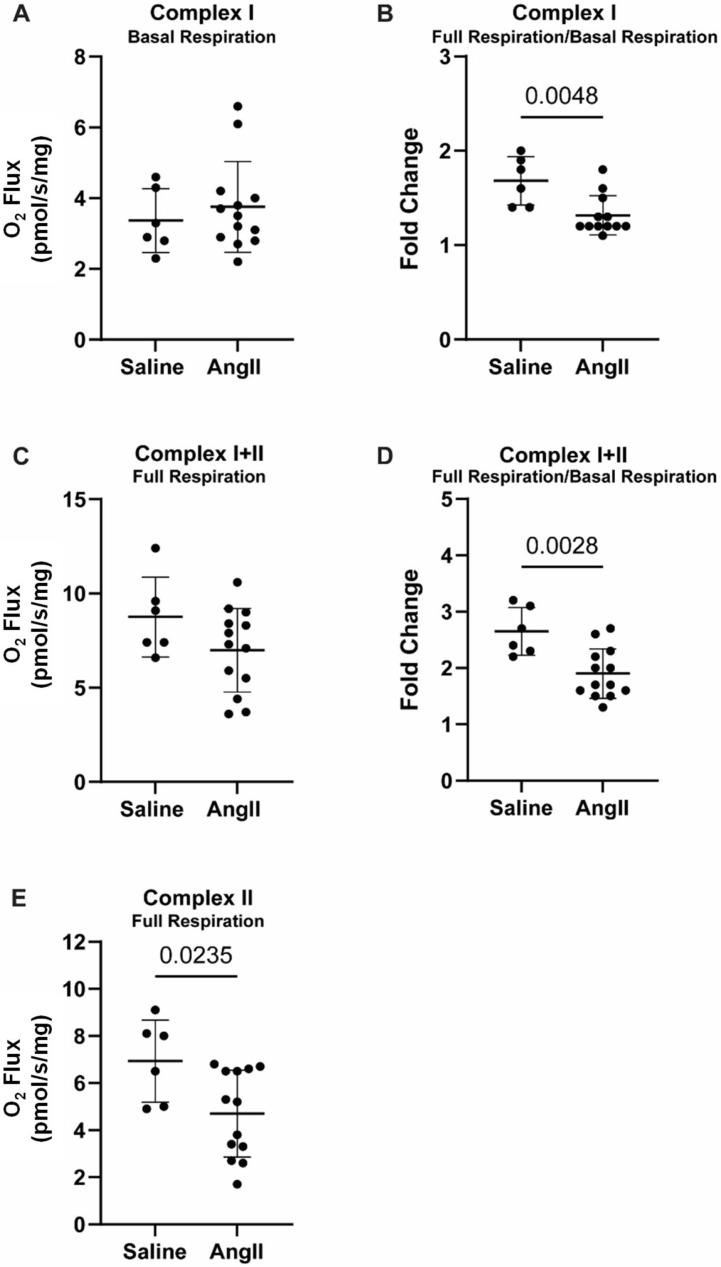
Mitochondrial respiration in the suprarenal aorta, normalized to aortic wet weight. (**A**) Complex I basal respiration; (**B**) Complex I full respiration/basal respiration ratio; (**C**) Complex I+II full respiration; (**D**) Complex I+II full respiration/basal respiration ratio; (**E**) Complex II full respiration. Data presented as mean ± standard deviation.

No significant differences in Complex I basal oxygen consumption were found in the suprarenal aorta (where AAA normally develops in this model) between angII-treated mice and saline controls (3.8 ± 1.29 vs. 3.4 ± 0.90 pmol/s/mg, Figure 3A). The increase (fold change) in Complex I oxygen consumption from basal respiration was significantly lower in aortas from angII-treated mice, compared to the saline controls (1.3 ± 0.21 vs. 1.7 ± 0.26, Figure 3B). No significant difference was found in Complex I+II oxygen consumption in aortas between the angII-treated mice and the saline control mice (7.0 ± 2.22 vs. 8.8 ± 2.12 pmol/s/mg, Figure 3C). However, relative to basal respiration, the increase in Complex I+II oxygen consumption was lower in aortas from the angII-treated mice than in the saline controls (1.9 ± 0.44 vs. 2.7 ± 0.42, Figure 3D). Complex II oxygen consumption was significantly lower in the angII-infused mice, compared to saline (4.7 ± 1.84 vs. 6.9 ± 1.75 pmol/s/mg, Figure 3E).

**Figure 4:**
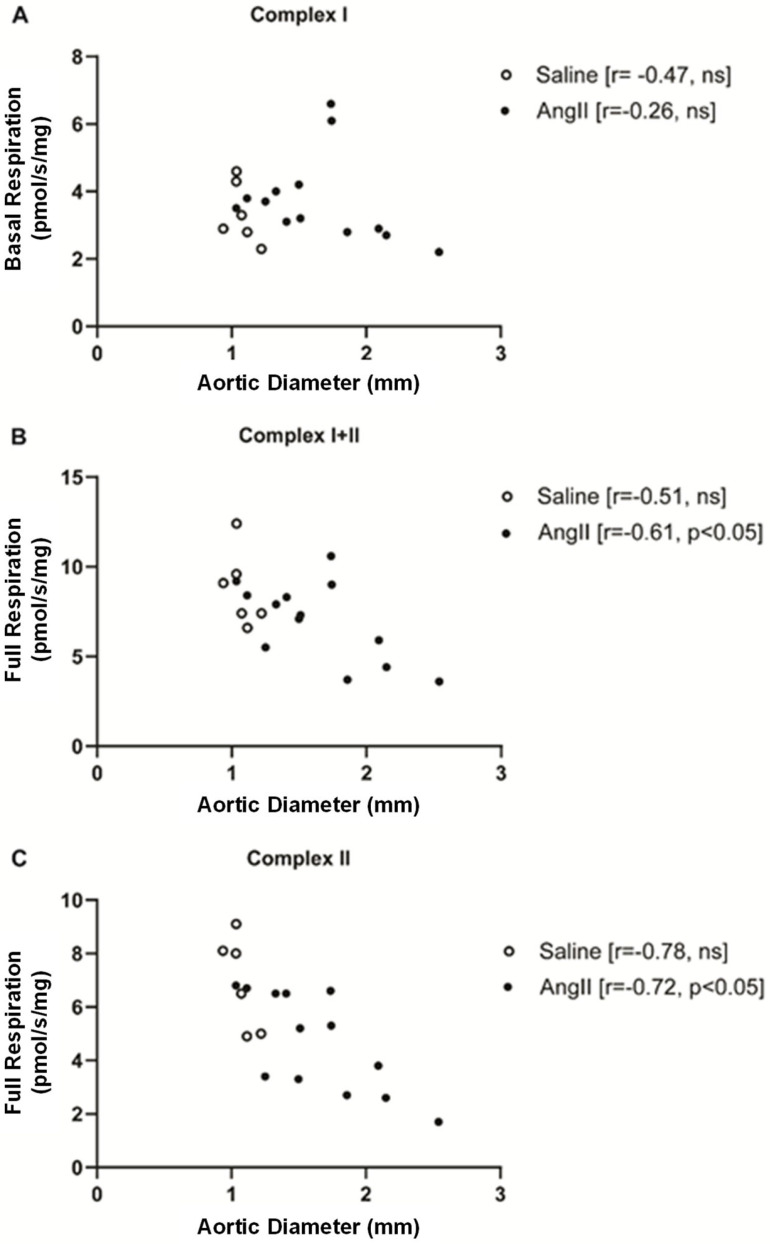
Correlation between mitochondrial functional capacity and aortic diameter. (**A**) Complex I basal respiration vs. aortic diameter; (**B**) Complex I+II full respiration vs. aortic diameter; (**C**) Complex II full respiration vs. aortic diameter.

Correlation analysis of oxygen consumption and aortic diameter showed that there was no change in mitochondrial respiration for Complex I basal respiration with aneurysm size (Figure 4A). However, for Complex I+II full respiration (Figure 4B) and Complex II full respiration (Figure 4C), the larger the aortic aneurysm, the lower the oxygen consumption. This was independent of duration of AngII exposure, which was 28 days for all subjects.

**Figure 5:**
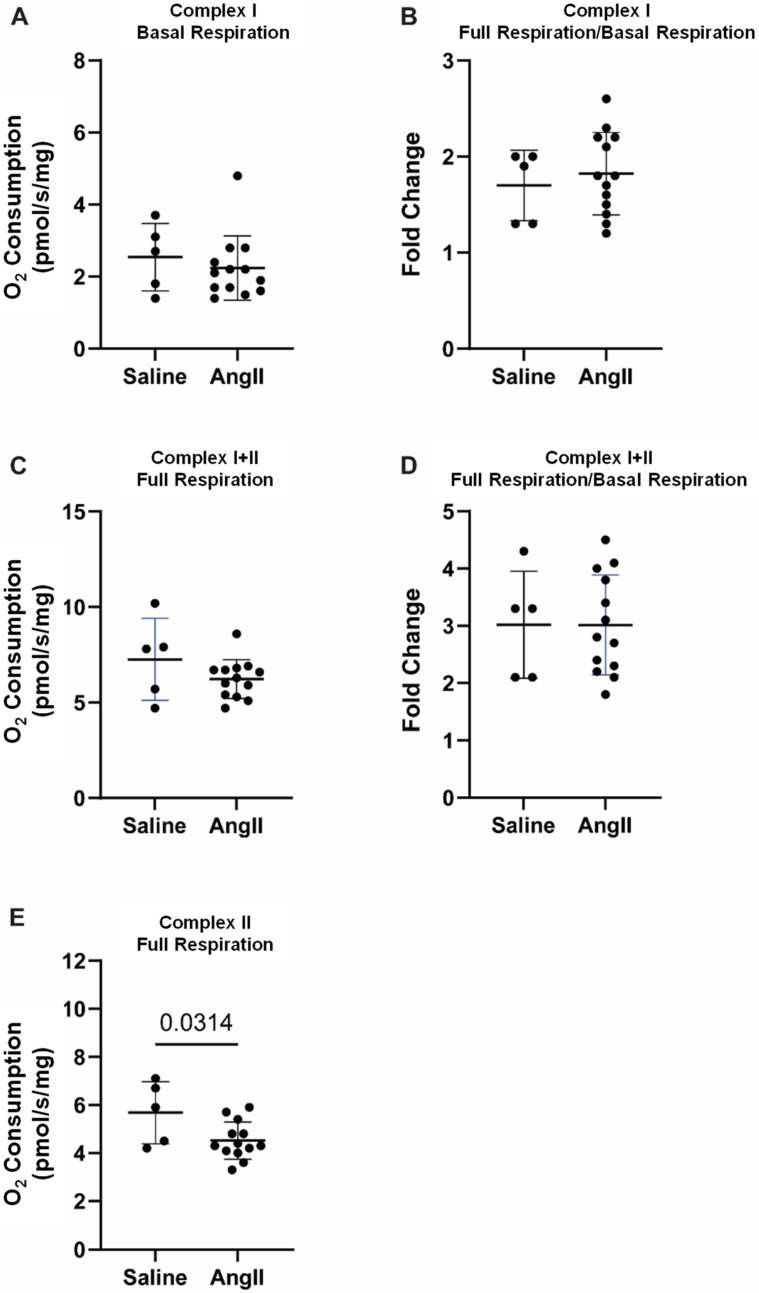
Mitochondrial respiration in the infrarenal aorta, normalized to aortic wet weight. (**A**) Complex I basal respiration; (**B**) Complex I full respiration/basal respiration ratio; (**C**) Complex I+II full respiration; (**D**) Complex I+II full respiration/basal respiration ratio; (**E**) Complex II full respiration. Data presented as mean ± standard deviation.

In the infrarenal aorta (where AAA normally does not develop in this model), no changes were observed in Complex I basal respiration (Figure 5A), Complex I full respiration (Figure 5B) or in Complex I+II full respiration (Figure 5C,D). Complex II full respiration was the only parameter analyzed that was affected in the infrarenal aorta, with the angII-treated mice having a lower oxygen consumption than the saline control (Figure 5E).

### 3.3. Effect of Rescue Treatment with Metformin in AAA Development 

**Figure 6:**
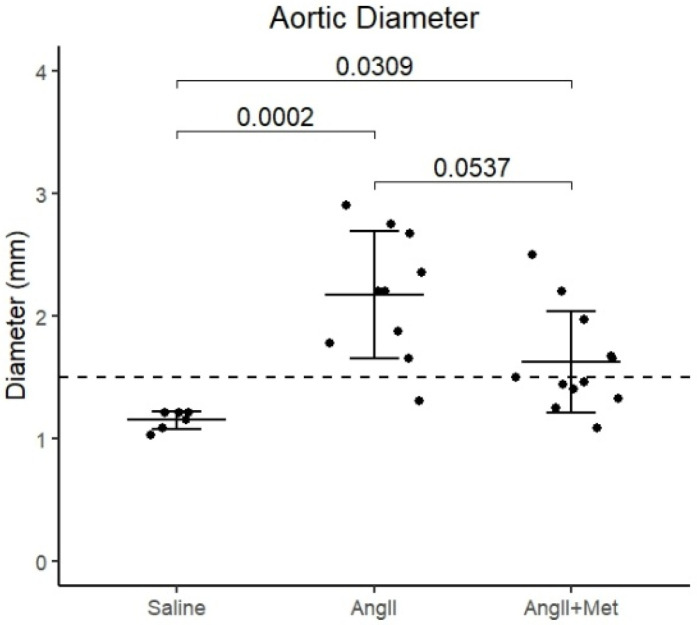
Aortic diameter measured by high-frequency ultrasound, on day 28 in the metformin rescue trial. Dotted line indicates threshold for aneurysm.

Previous experiments in angII-infused mice have shown that metformin given on the same day as angII infusion prevents aneurysm formation. In this study we also examined if treatment with metformin after seven days of angII infusion could inhibit disease development. All control mice survived until experimental endpoint. In the angII-treated groups, 10 out of 32 mice died from dissection or rupture, in the first six days from disease induction (before beginning of metformin treatment). For the surviving mice, aortic diameter was significantly increased in the angII-treated group (2.2 ± 0.52 mm) and angII+metformin (1.6 ± 0.41 mm)-treated group, compared to saline (Figure 6). A trend to a decreased aortic diameter in the angII+metformin group compared to angII was observed (*p* = 0.0537). Treatment with metformin did not affect blood glucose levels or weight at termination (Table 2).

### 3.4. Metformin Does Not Affect Mitochondrial Function of the Aorta

**Figure 7:**
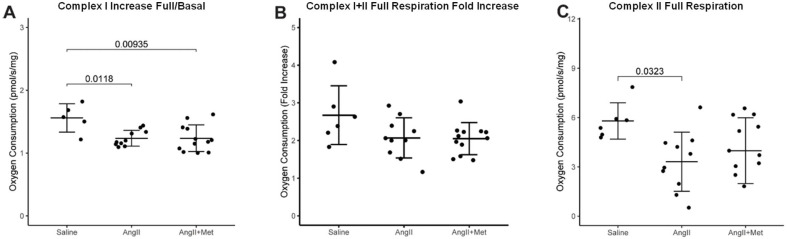
Mitochondrial respiration in the suprarenal aorta (normalized to aortic wet weight), for the rescue trial with metformin (**A**) Complex I full respiration/basal respiration ratio; (**B**) Complex I+II full respiration/basal respiration ratio; (**C**) Complex II full respiration. Data presented as mean ± standard deviation.

The mechanism of metformin on preventing aneurysm development is not known. Treatment of mice with metformin after one week of angII infusion up to 4 weeks was performed to investigate if any of the effects of metformin on aneurysm growth is mediated through mitochondria functional capacity. However, rescue treatment did not affect or restore mitochondrial function. For Complex I, fold increase from basal to full respiration was higher in the saline group (1.6 ± 0.22), compared to the angII-treated group, confirming what we previously observed. However, no difference was found between the angII and angII+metformin groups (1.2 ± 0.12 vs. 1.2 ± 0.21, Figure 7A). No significant differences in Complex I+II fold increase from basal to full respiration were found between the saline group, angII group and angII-treated group (2.7 ± 0.78 vs. 2.1 ± 0.53 vs. 2.0 ± 0.43, Figure 7B). Complex II full respiration was lower in the angII-treated group, compared to saline (3.3 ± 1.80 vs. 5.8 ± 1.11 pmol/s/mg). No differences were found with the angII+metformin group (4.0 ± 2.00 pmol/s/mg, Figure 7C).

## 4. Discussion

The pathophysiological mechanisms of AAA development are not yet completely understood. Consequently, the mechanisms through which metformin prevents its development are also not fully elucidated. To better understand these mechanisms, methods that allow functional studies of the aneurysm as a whole entity are needed. In this paper, we aim to optimize a method to analyze mitochondrial functional capacity in intact aneurysmal aortas, to better understand the effects of mitochondrial function on disease development.

Measurement of mitochondrial function in permeabilized tissues is preferred, compared to other preparations such as isolated mitochondria or permeabilized cells, as it allows to measure OXPHOS in circumstances most similar to physiological conditions. Ex vivo measurement of mitochondrial function in aortic tissue has been previously performed as follows: Feely et al. measured mitochondrial function using 3 mm longitudinally cut aortic rings (without permeabilization) and measured aortic function by seahorse XF-24 protocol [10]; Docherty et al. measured mitochondrial function using 10 mg aortic tissue samples, previously permeabilized with saponin for 15 min, by HRR [11]; Galambo et al. measured aortic function by pooling two aortas per sample (6–8 mg), after permeabilization in saponin for 30 min, by HRR [12]; Docherty et al. have also applied this method to human atherosclerotic tissue samples [13]. However, analysis of mitochondrial function in whole permeabilized aneurysmal tissue has not been reported. The dose of digitonin in our setup was chosen based on initial dose–response experiments in which mitochondrial integrity in the tissue was checked by cytochrome c in HRR-experiments. Also, pathologically altered tissues was checked this way and was found non-responsive to cytochrome c.

AAA is a complex disease and despite the efforts to discover its pathological mechanisms, there is a low success rate in translating pre-clinical findings into clinical advancements. The focus on individual cell types rather than the whole aneurysm as one entity could be one of the translational obstacles. For this reason, we evaluated mitochondrial functional capacity in permeabilized whole aneurysmal tissue and confirmed that, as predicted by genome analysis [4], mitochondrial function is impaired in aneurysmal tissue.

Our results show that basal respiration is not affected by angII treatment and aneurysm development, thus healthy and aneurysmal aortas start at the same level of oxygen consumption. However, when ADP is provided, stimulating the production of ATP by the ATP synthase and thus increasing oxygen consumption to maintain the transport of H^+^ ions to the intermembrane space, we observe that aortas from angII-treated mice have a lower capacity to increase oxygen consumption. Same effect is observed with the addition of succinate to promote full respiration, supported by Complexes I and II. Evaluation of Complex II full respiration once again shows a decreased respiration in angII-treated tissues. Mitochondrial function is impaired in the angII-treated mice, as these aortas have a reduced respiration capacity. Furthermore, we observed that mitochondrial functional capacity is negatively correlated to aneurysm size and progression of AAA is accompanied by deterioration of mitochondrial function. Mitochondrial activity is associated with aortic wall stability and aneurysm development. By restoring VSMC mitochondrial function, AAA is attenuated in mice [28] and Marfan syndrome patients have significant reduction in key regulators of mitochondrial function and impaired mitochondrial respiration in VSMC from these patients is associated with pathological switching in loss of contractile phenotype and increased extracellular matrix remodeling [29,30].

The effect on mitochondrial functional capacity seems to be localized to the suprarenal aorta, as only Complex II-supported respiration was affected in the infrarenal aorta. This indicates a functional change that does not lead to an impairment of Complex I and II full respiration and ATP production. Further exploration of mitochondrial function could clarify why aneurysms develop suprarenal and not infrarenal in the angII mouse model.

Treatment with metformin, though potentially reducing the formation of AAA, does not restore mitochondrial function. The parameters altered in the previous experiment (fold increase in Complex I and Complex I+II respiration and Complex II full respiration) did not differ between angII and angII+metformin groups, showing that metformin was unable to revert the loss of mitochondrial functional capacity in this set-up.

In this study, we could not confirm the effect of metformin on AAA development, although we did observe a tendency for decreased aneurysm formation. We have previously shown that treatment with metformin in drinking water reduces AAA formation in the angII model [21]. In that paper, the average aortic diameter in the angII+metformin group is slightly larger than 1.5 mm, similar to our results in the rescue trial. With this, we can infer that metformin reduces AAA formation, although the efficacy of treatment decreases in a rescue style setting. Nonetheless, others have shown that in the elastase model, a rescue trial with metformin (starting 4 days after disease induction) does prevent AAA growth [20]. It is notable that in that paper the dose of metformin used is larger and administered by oral gavage. Wang and colleagues showed that clinically relevant concentrations of metformin increase mitochondrial respiration while supra-pharmacological concentrations halted mitochondrial respiration [24].

In this study, we started treatment with metformin seven days post disease induction. The literature indicates that one week after disease induction, diameter in the angII model can increase up to 50% [31,32]. We decided on this set-up, as it has not been evaluated before in the angII-infused model and we believe that the results have a higher potential to translate to the clinical reality, as in humans, treatment starts after the initiation of the disease.

## 5. Conclusions

In conclusion, we demonstrate, for the first time, that mitochondrial functional capacity can be evaluated in murine whole aneurysmal tissue, providing a method for a physiological approach to the study of mitochondrial function in AAA. Furthermore, we prove that mitochondrial functional capacity is impaired in AAA, as predicted by genome analysis. However, rescue treatment with metformin is not sufficient to recover mitochondrial function and seems, at least partly, not to be the mechanism behind prevention of aneurysm.

## Figures and Tables

**Table 1:** Survival rate and aneurysm formation in the AngII-induced mouse model.

	Saline (*n* = 6)	AngII (*n* = 13)	*p*-Value
Survival	6/6 (100%)	13/20 (65%)	ns
Aortic diameter (mm)	1.1 ± 0.09	1.6 ± 0.44	*p* < 0.05
Suprarenal wet weight (mg)	1.7 ± 0.80	11.1 ± 7.62	*p* < 0.05
Infrarenal wet weight (mg)	3.0 ± 1.36	6.1 ± 1.14	*p* < 0.05

*n* = number of mice included in each group. ns = non-significant.

**Table 2:** Survival rate, aneurysm formation, blood glucose and body weight in the metformin rescue trial.

	Saline (*n* = 6)	AngII (*n* = 10)	AngII+met (*n* = 12)	*p*-Value
Survival	6/6 (100%)	10/16 (62.5%)	12/16 (75%)	ns
Body weight (g)	29.4 ± 2.10	29.2 ± 1.93	28.7 ± 1.82	ns
Blood glucose (mmol/L)	8.6 ± 1.85	7.6 ± 2.10	8.7 ± 2.19	ns
Suprarenal wet weight (mg)	1.9 ± 0.41	38.6 ± 45.39	12.9 ± 10.47	* *p* < 0.05 # *p* < 0.05

* Saline vs. AngII. # Saline vs. AngII+Met. ns = non-significant.

## Data Availability

The original contributions presented in this study are included in the article. Further inquiries can be directed to the corresponding author.
